# Fluorescence Hyperspectral Imaging of Oil Samples and Its Quantitative Applications in Component Analysis and Thickness Estimation

**DOI:** 10.3390/s18124415

**Published:** 2018-12-13

**Authors:** Wentao Jiang, Jingwei Li, Xinli Yao, Erik Forsberg, Sailing He

**Affiliations:** National Engineering Research Center of Optical Instrumentation, Centre for Optical and Electromagnetic Research, Zhejiang University, Hangzhou 310058, China; 21630007@zju.edu.cn (W.J.); 11430020@zju.edu.cn (J.L.); 21760141@zju.edu.cn (X.Y.); erikf@zju.edu.cn (E.F.)

**Keywords:** fluorescence hyperspectral imaging, oil detection, principal component analysis, *K*-means clustering

## Abstract

The fast response and analysis of oil spill accidents is important but remains challenging. Here, a compact fluorescence hyperspectral system based on a grating-prism structure able to perform component analysis of oil as well as make a quantitative estimation of oil film thickness is developed. The spectrometer spectral range is 366–814 nm with a spectral resolution of 1 nm. The feasibility of the spectrometer system is demonstrated by determining the composition of three types of crude oil and various mixtures of them. The relationship between the oil film thickness and the fluorescent hyperspectral intensity is furthermore investigated and found to be linear, which demonstrates the feasibility of using the fluorescence data to quantitatively measure oil film thickness. Capable of oil identification, distribution analysis, and oil film thickness detection, the fluorescence hyperspectral imaging system presented is promising for use during oil spill accidents by mounting it on, e.g., an unmanned aerial vehicle.

## 1. Introduction

The frequency of oil spill accidents continues to grow [1,2], doing great harm to marine eco-systems. Oil spills negatively impact human health by contaminating food from the oceans [3,4,5] and hinder the development of coastal economies [6]. Hence, automatic oil spill detection systems are of great importance. Presently, several methods such as gas chromatography [7,8], remote sensing with a reflectance spectrum [9,10], and fluorescence spectroscopy [11,12,13] are utilized to analyze oil samples. Using gas chromatography, oil types can be identified by dividing the characteristic alkanes from the sample [14]. Bruflodt et al. used two-dimensional gas chromatography to perform oil sample type classification and quantitative comparison between oil samples [15]. While having the advantages of high sensitivity and strong selectivity, gas chromatography is not practical for real-time ocean applications due to bulky instrument size and lengthy analysis times. In remote sensing, passive sensors are used to collect the reflective spectra of objects illuminated by sunlight. Using remote sensing, the information of a large area can be obtained [16]. However, the method is limited by weather conditions and does not work at night, unless an expensive long-wave infrared hyperspectral sensor is used. Fluorescence spectroscopy is another effective method for oil sample analysis in which oil samples excited by a laser can emit a fluorescence signal that can be used to identify the type of oil. The intensity of fluorescence is furthermore related to the thickness of the oil film [14]. Hou et al. designed and set up a system, using a xenon lamp as the light source and a photomultiplier tube (PMT) as the detector, which can detect and monitor coastal oil spills in real time [17], but cannot identify the type of oil. Hyperspectral laser-induced fluorescence LiDAR (light detection and ranging) was developed for oil detection in open and coastal waters [18,19]. A pulsed excimer laser with a 308 nm wavelength was utilized to perform raster scanning of the waters. Effective as the method was, it is challenging to achieve fast, high-throughput detection with a compact structure. 

In this work, we designed and fabricated a cost-effective compact fluorescent hyperspectral detection system capable of component analysis and thickness estimation of oil samples. A 405 nm line laser is used to excite the fluorescence of oil samples, and an imaging spectrometer fabricated in-house is utilized as a sensor to detect the sample spectra. With a line laser as the excitation source and an imaging spectrometer as the sensor, fast and high-throughput detection can be achieved. We also performed oil film thickness estimation by using diesel oil films with different thicknesses. A linear relationship between the fluorescence intensity and thickness of the diesel oil film was observed. A trial test in open space of this hyperspectral detection system was conducted in the laboratory.

## 2. Materials and Methods

### 2.1. Design of the Fluorescent Hyperspectral Detection System

**Figure 1:**
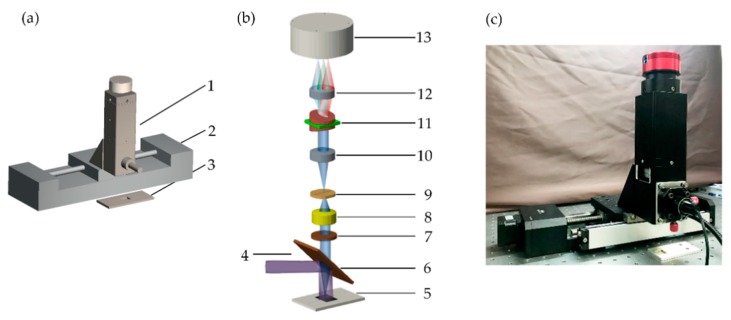
(**a**) Schematic of the fluorescent hyperspectral detection system. 1, Imaging spectrometer; 2, motorized linear stage; 3, sample slot. (**b**) Optical path of the hyperspectral detection system. 4, Line laser; 5, sample slot; 6, dichroic beam splitter; 7, longpass filter; 8, lens; 9, slit; 10, aspherical achromatic lens; 11, prism-grating-prism pair; 12, aspherical achromatic lens; 13, area-array complementary metal-oxide-semiconductor (CMOS) camera. (**c**) A photo of our hyperspectral detection system.

Figure 1a shows a schematic diagram of the fluorescent hyperspectral detection system. The system consists of an imaging spectrometer fabricated in-house, a motorized linear stage, and a sample slot. Figure 1b shows the optical path of the system, the details of which are described in the following. A 405 nm line laser (giving a light-sheet output with a line excitation on the sample) with 200 mW power was used as the excitation source, which, after being reflected by a 425 nm dichroic beam splitter (DMLP425R, Thorlabs, Newton, NJ, USA), excites the fluorescence signal from the sample in the sample slot. The fluorescence signal will pass through the dichroic beam splitter, and a longpass filter (FELH0450, Thorlabs, Newton, NJ, USA) and is then focused onto a 10 μm slit of the imaging spectrometer through an imaging lens (AC254-030-A, Thorlabs, Newton, NJ, USA). After passing through the slit, the fluorescence is firstly collimated by an aspherical achromatic lens (#49-665, Edmund, Barrington, NJ, US) and then dispersed by a prism-grating-prism structure. The parameters of the grating are 300 grooves per millimeter with a 17.5° groove angle (GT25-03, Thorlabs, Newton, NJ, USA). Finally, the signal is focused on an area-array complementary metal-oxide-semiconductor camera (ASI74MM, ZWO, Suzhou, China) by another aspherical achromatic lens (#49-665, Edmund). The resolution of the CMOS camera is 2.35 megapixels (1936 × 1216) with a peak quantum efficiency of 78% at 530 nm. The long axis of the camera was set as the spatial axis, while the short axis was set as the spectral axis. The imaging spectrometer was mounted on the motorized linear stage. Through driving the motorized linear stage, the fluorescence hyperspectral data was obtained. Figure 1c shows a photo of the hyperspectral detection system.

**Figure 2:**
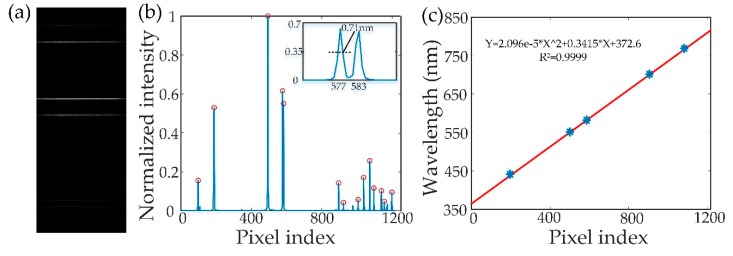
Calibration of the in-house fabricated spectrometer with a calibration source (HG-1 calibration source, Ocean Optics). (**a**) Calibration image. (**b**) Peak distribution. (**c**) The calibration curve.

The spectral calibration of the imaging spectrometer was performed by using a calibration source (HG-1 calibration source, Ocean Optics, Largo, FL, USA). Figure 2a shows an image captured by the area-array camera while the calibration source illuminated the slit without passing the longpass filter. The vertical direction is the axis of the wavelength, while the horizontal direction is the spatial distribution of the line fluorescence. Figure 2b shows the spectrum of the calibration source in the area-array camera, in which the wavelength of the light source is related with the pixel index of the camera. The inset of Figure 2b shows the peak of wavelength of 576.960 nm with 0.71 nm spectral resolution. The wavelengths of the calibration source at 435.833 nm, 546.074 nm, 576.960 nm, 696.543 nm, and 772.376 nm and their corresponding spatial positions are used in the curve fitting process [20] by a least squares polynomial fit method. The result of the wavelength calibration is shown in Figure 2c.

### 2.2. Oil Samples and Spectral Data Collection

**Figure 3:**
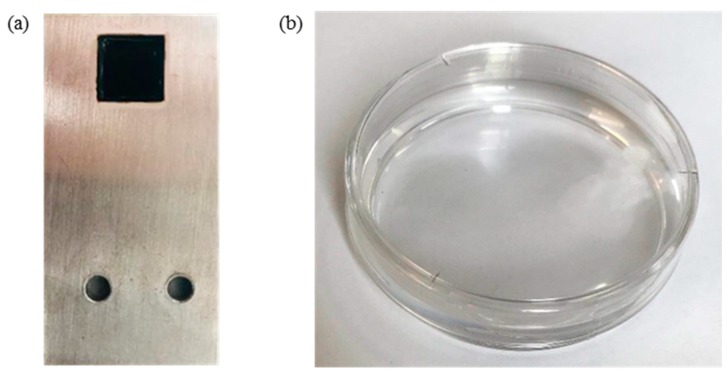
(**a**) Photo of the sample slot for crude oil type detection. (**b**) Photo of the sample cell for thickness detection of diesel oil.

The spectra of three types of crude oil were measured, and the spatial distribution of various mixed sample compositions of the three crudes (listed in Table 1) was analyzed. Figure 3a shows a photo of the sample slot for type classification of crude oil. 

Thickness measurements of diesel oil were furthermore made, in which diesel oil with varying sample volume was added to the surface of water (6 mm deep) in a Petri dish (Figure 3b). The volume of diesel *V* was measured precisely by using a pipette, and the thickness *h* of the diesel oil was calculated by the volume of diesel oil *V* over the area of the container *S*:

(1) h=V/S.

Diesel oil with thickness in the range from 100 μm to 400 μm at 25 μm intervals was used for thickness estimation and was used to mimic an oil spill accident with a density of over 50,000 liters per square kilometer [21]. Figure 3b shows a photo of the sample cell filled with diesel oil for thickness detection of the diesel oil. It is worth mentioning that diesel oil cannot be recognized in water by the naked eye.

### 2.3. Data Analysis

For the hyperspectral data, in order to eliminate the effect of dark currents and ambient light, background subtraction was made. A dark background was obtained by measuring the samples without exciting light. Then, the background was subtracted from the hyperspectral data. We also used the method in [22] to eliminate the stripe noise of the hyperspectral data caused by the unevenness of the slit and nonlinear effects between camera pixels [23]. At the end of the data preprocessing, Savitzky-Golay smoothing was utilized [24]. To analyze the hyperspectral data of the crude oil samples after preprocessing, principal component analysis (PCA) and *K*-means clustering were used. We utilized PCA to reduce the dimensionality of the hyperspectral data in order to speed up the computing process. PCA is a common method for the dimensionality reduction of spectral data [25,26]. The procedures for PCA are briefly summarized in this section.

Nonlinear iterative partial least squares is an efficient method used to implement PCA [27]. Assume that *X* is the mean-centered data of hyperspectral data. *E*_(0)_, set equal to *X*, is the matrix used to calculate the zeroth PC (*PC*_0_). The *t* vector is set to a column in *X*, and *t^T^t* will be the scores for *PC_i_*. The *p* vector will be the loadings for *PC_i_*. *E*_(*i*−1)_ is projected to *t* to find the corresponding loading *p*:

(2) $$p=\frac{E_{\left(i-1\right)}^{T}t}{t^{T}t}\;.$$

Then, we normalize loading vector *p* to length 1:

(3) $$p=p*{\left(p^{T}p\right)}^{-0.5}.$$

Next, we project *E*_(*i*−1)_ onto *p* to find the corresponding score vector *t*:

(4) $$t=\frac{E_{\left(i-1\right)}^{T}p}{p^{T}p}\;.$$

If the difference between $\tau_{new}=t^{T}t$ and $\tau_{old}$ (from the last iteration) is larger than the threshold, we return to Equation (2) and do another iteration of the computations from Equation (2) to Equation (4). Finally, we remove the estimated PC component from *E*_(*i*−1)_ and obtain the matrix to calculate the next PC:

(5) $$E_{\left(i\right)}=E_{\left(i-1\right)}-\left(tp^{T}\right).$$

Using this method, the first few principle components with highest scores can be calculated. Then, *K*-means clustering was applied to divide all the spectra into *K* categories, where *K* is the number of types of oil in the sample [28]. Assume that *x*_1_, *x*_2_, …, *x_i_* are the spectral signatures of the data, that *S*_1,_ *S*_2_, …, *S_k_* is the set of clusters, and that *m*_1_, *m*_2_, …, *m_k_* are randomly selected as the initial centroids of each cluster. We assign each spectral signature to the cluster which has the least squared Euclidean distance from the centroid to the spectral signature:

(6) $$S_{i}=\left\{x_{p}:{\left|\left|x_{p}-m_{i}\right|\right|}^{2}\leq {\left|\left|x_{p}-m_{j}\right|\right|}^{2}\;\forall j,\;1\leq j\leq k\right\}$$

where each *x_p_* is assigned to *S_i_*.

Then, we update the centroids by calculating the means of every cluster:

(7) $$m_{i}=\frac{\sum_{x_{j}\in S_{i}}x_{j}}{\left|S_{i}\right|}\;.$$

If the difference between before and after the update of *m_i_* is smaller than the threshold, the clustering ends; otherwise, we repeat Equations (6) and (7). Finally, the distribution of the different crude oils can be obtained by marking the positions of the spectra of different types with different colors. 

To estimate the thickness of diesel oil, the relationship between oil film thickness and fluorescence intensity was acquired by measuring the fluorescence hyperspectral data of diesel oil films with different thicknesses. The fluorescence intensity of samples with different thicknesses was calculated by averaging all the spectral data measured from the same thickness to reduce the error caused by uneven distributions of oil films. By fitting the fluorescence intensity and the thickness of the oil film, the relationship between them was obtained.

## 3. Results and Discussion

### 3.1. Type Classification and Distribution Analysis

**Figure 4:**
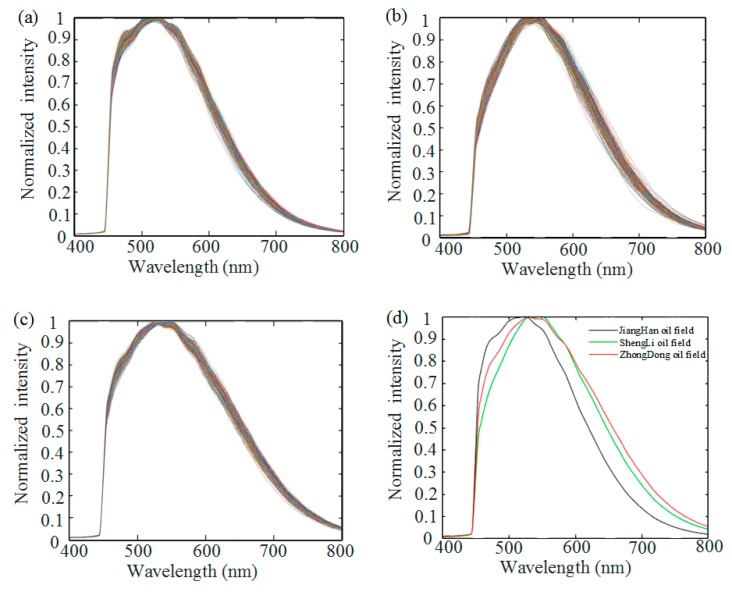
Normalized spectra of three types of crude oils. (**a**) Crude Oil 1. (**b**) Crude Oil 2. (**c**) Crude Oil 3. (**d**) The averaged normalized spectrum of the three types of crude oil.

The seven samples listed in Table 1 were used for type classification and distribution analysis. By scanning the sample with the motorized linear stage, the hyperspectral data of the sample were obtained. The sample stage was moved at a speed of 20 mm/s, and a picture with an exposure time of 100 ms was captured every 50 μm. In total, 400 pictures were captured for each sample. Figure 4 shows the normalized spectra and averaged normalized spectra of the three types of crude oil at each pixel. As can be seen in Figure 4a–c, the spectra of different types of crude oil are close to identical at different spatial positions. However, as can be seen in Figure 4d, the averaged normalized spectra of the three types of crude oil are quite different from each other, indicating the possibility to identify the types of oil samples through fluorescence. The peak wavelength of Crude Oil 1 is around 510 nm, which is shorter than those of the other two samples, and the normalized fluorescence intensity of Crude Oil 3 is larger than that of Crude Oil 2 at around 525 nm. These features can be combined to determine the type of crude oil. It should be noticed that these features were obtained without the intensity calibration, after which they can be seen more clearly.

Prior to analyzing the data of the mixed samples, PCA was applied to reduce the dimension of the spectral data as discussed in the previous section. The number of dimensions was decided by the contribution of each component. Table 2 presents the contribution of the component obtained from the mixed samples, Samples d, e, f, and g. Following the dimensionality reduction, two dimensions were determined for Samples d, e, and f and three dimensions for Sample g. 

**Figure 5:**
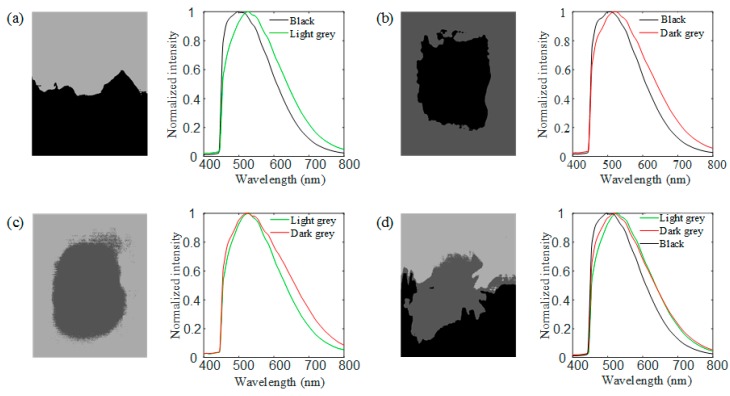
Imaging (pseudo color) of the spatial distribution of crude oils in four mixed samples and the average spectra of the different regions for (**a**) a mixed sample of Crude Oil 1 and Crude Oil 2; (**b**) a mixed sample of Crude Oil 1 and Crude Oil 3; (**c**) a mixed sample of Crude Oil 2 and Crude Oil 3; and (**d**) a mixed sample of Crude Oil 1, Crude Oil 2, and Crude Oil 3.

Following this, *K*-means clustering was applied for each pixel. Pixels of the same type were marked with the same color, yielding spatial distributions as shown in Figure 5. Regions marked with different colors represent different types of crude oil. Black color represents Crude Oil 1, light gray color represents Crude Oil 2, and dark gray represents Crude Oil 3. Referring to the sample composition descriptions in Table 1, we can conclude that the analysis of the spatial distribution of samples is correct.

### 3.2. Thickness Estimation of Diesel Oil 

**Figure 6:**
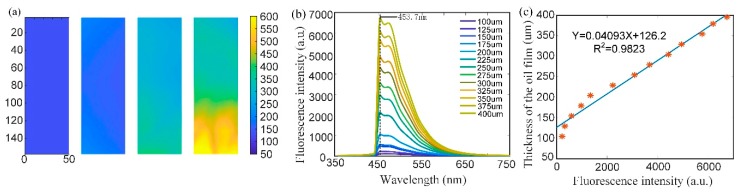
The relation of the thickness of the diesel film and its fluorescence intensity. (**a**) The distributions of the thicknesses of oil with average thicknesses of 100 μm, 200 μm, 300 μm, and 400 μm; (**b**) The spectra of the different thicknesses of the oil film. (**c**) The fitting curve of the thickness of the oil film and fluorescence intensity of the peaks of the spectra.

Diesel oil with thicknesses in the range from 100 μm to 400 μm at 25 μm intervals was used for thickness estimation. The required volume of diesel was drawn by using a pipette, and the thickness of each sample was calculated by dividing the volume of diesel oil by the surface area of the container, which has a diameter of 5.36 cm. In order to scan a large region in a short time, 50 pictures with an exposure time of 100 ms were captured for each sample at spatial intervals of 0.5 mm. Figure 6 shows the relationship between the thickness of the diesel oil film and the fluorescence intensity. Figure 6a shows the distributions of the thickness of the oil with average thicknesses of 100 μm, 200 μm, 300 μm, and 400 μm (quite nonuniform). Figure 6b shows the mean fluorescence spectra of oil films with different thicknesses. Each spectrum was obtained by averaging all the pixels in the sample. Figure 6c is the result of linear regression analysis of the intensity of peaks of the spectra versus the thickness of the diesel film. A linear relationship with coefficient of determination R^2^ equal to 0.9823 was obtained, which shows the feasibility of the oil thickness estimation.

### 3.3. Trial Test in Open Space

**Figure 7:**
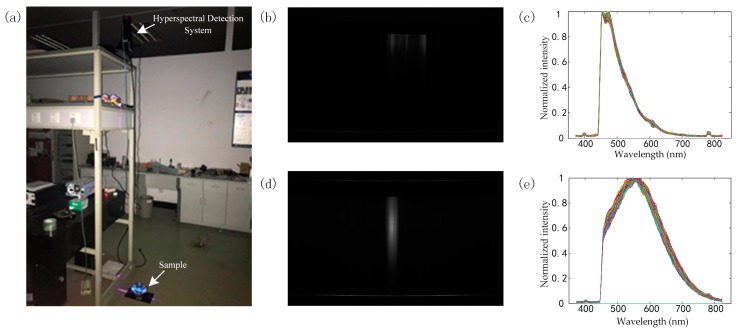
(**a**) The environment of the experiment. (**b**) An image of the fluorescence line of diesel oil, as captured by the camera. (**c**) Normalized spectra of the fluorescence line of diesel oil. (**d**) An image of the fluorescence line of Crude Oil 1, as captured by the camera. (**e**) Normalized spectra of the fluorescence line of Crude Oil 1.

In order to test the feasibility of the system in open space, we made a trial test in the laboratory. The fluorescent hyperspectral detection system was put above a 2.2 m high shelf while the sample was on the ground, as shown in Figure 7a. The samples chosen were Crude Oil 1 with an exposure time of 10 s and diesel oil with an exposure time of 3 s. It can be seen, though with limited excitation power (200 mW) and a cheap CMOS camera, that the system was able to obtain spectra of high quality. Thus, we believe that the system has the potential to be used in detection in open space.

## 4. Conclusions

We presented a fluorescence imaging system and used it to analyze the types and distributions of mixed crude oil samples as well as the thickness of a diesel oil film. The system consists of an imaging spectrometer fabricated in-house that has a spectral range of 366–814 nm and a spectral resolution of about 1 nm. The picture acquisition time is about 100 ms. The speed of the hyperspectral data acquisition can be increased by enhancing the power of the excitation light and the sensitivity of the camera. The lateral spatial resolution is determined by the pixel size of the camera, the focal length of the imaging lens, and the distance from the sample slot to the lens [29,30], while the vertical spatial resolution is determined by the step size of the motorized linear stage. Hyperspectral data were obtained by scanning the sample through moving the imaging spectrometer using the motorized linear stage. PCA and *K*-means clustering were used to analyze the mixed crude oil data, after which the crude oil types and spatial distributions of the mixed crude oil samples could be obtained. We furthermore demonstrated thickness estimation for diesel oil samples. A linear relationship between the intensity of peak of spectra and the thickness of the diesel film was obtained, with coefficient of determination R^2^ equal to 0.9823. We also made a trial test of the hyperspectral detection system in the laboratory by acquiring the spectra of oil samples. The experimental results above show the capability of fluorescence hyperspectral imaging in the quantitative analysis of oil spills.

By using a laser with higher power intensity and a camera with higher sensitivity, our fluorescence imaging system could detect oil films thinner than 100 μm, giving it the potential to be used in practical complex field analysis of oil spill accidents. The system, however, is limited to the samples that can be excited and emit fluorescence. It may also be interfered with by components in seawater that can emit fluorescence. By combining the system with, e.g., an unmanned aerial vehicle [31,32], this system has the potential to be used in response to oil spill accidents.

## Figures and Tables

**Table 1:** Types of crude oil contained in the sample.

Sample Label	Type of Oil	Add Location
a	Crude Oil 1	
b	Crude Oil 2	
c	Crude Oil 3	
d	Crude Oil 1 and Crude Oil 2	Crude Oil 2 (up*); Crude Oil 1 (down*)
e	Crude Oil 1 and Crude Oil 3	Crude Oil 1 (inner*); Crude Oil 3 (outer*)
f	Crude Oil 2 and Crude Oil 3	Crude Oil 3 (inner*); Crude Oil 2 (outer*)
g	Crude Oil 1 and Crude Oil 2 and Crude Oil 3	Crude Oil 2 (up*); Crude Oil 3 (middle*); Crude Oil 1 (down*)

* up, middle, down, inner, and outer represent the relative position of the crude oil in the sample slot.

**Table 2:** Contribution of principal components for four different mixed samples.

Principle Component	Sample d	Sample e	Sample f	Sample g
1	3.29426	3.09946	0.11511	2.64265
2	0.01213	0.04963	0.06621	0.17782
3	0.00535	0.01946	0.00703	0.00876
4	0.00252	0.00567	0.00393	0.00595
5	0.00130	0.00168	0.00071	0.00235
6	0.00057	0.00061	0.00062	0.00070
